# Data on the stem cells paracrine effects on apoptosis and cytokine milieu in an experimental model of cardiorenal syndrome type II

**DOI:** 10.1016/j.dib.2018.10.127

**Published:** 2018-11-01

**Authors:** Giorgio Vescovo, Chiara Castellani, Marny Fedrigo, Grazia Maria Virzì, Giovanni Maria Vescovo, Regina Tavano, Michela Pozzobon, Annalisa Angelini

**Affiliations:** a Internal Medicine, S. Antonio Hospital, Padova, Italy; b Dept. Cardiac, Thoracic, Vascular Sciences, and Public Health, University of Padua, Padova, Italy; c Department of Nephrology, Dialysis and Transplant, San Bortolo Hospital, Vicenza, Italy; d IRRIV-International Renal Resarch Institute of Vicenza, Italy; e Dept. Biomedical Sciences, University of Padua, Padova, Italy; f Dept. Women and Children Health, University of Padua, Padova, Italy; g Institute of Pediatric Research Città della Speranza, Padova, Italy

## Abstract

The data reported in this article are related to the paper entitle “Stem cells transplantation positively modulates the heart-kidney cross talk in Cardiorenal Syndrome Type II” (Vescovo et al., 2019), which analyzed the impact of stem cells injection in cardiorenal syndrome type II. The dataset contains detailed information on apoptosis and cytokines milieu modification after injection of c-Kit–selected human amniotic fluid stem cells (hAFS) or rats vascular progenitor cells (rSVC-GFP group) in an experimental model of CRSII. The data can be useful for clarifying the paracrine effects exerted by the injected cells.

**Specifications table**

**Table:** 

Subject area	*Biology and medicine*
More specific subject area	*Regenerative Medicine*
Type of data	*Table, graph and figures*
How data were acquired	*The authors acquired the data using two methods:*
	*-Zeiss microscope optical microscope connected to a computer via a video*
	*camera (JVC 3-CCD, Yokohama, Japan) and software for*
	*image analysis (Image PRO-Plus 4.0; Media Cybernetics, Silver Spring, MA).*
	*-Bioplex suspension array system (Bio-Rad, Milan, Italy)*
Data format	*Raw and analyzed*
Experimental factors	*Data acquired were analyzed with graphpad statistical software and plotted in the graph*
Experimental features	*The data were collected by analysis of TUNEL and ELISA technique experiments on rat kidney tissue and rat serum of the different experimental animal groups*
Data source location	*University of Padova, Italy*
Data accessibility	*Title: Stem cells transplantation positively modulates the heart-kidney cross talk in Cardiorenal Syndrome Type II*
	*Journal: International Journal of Cardiology**[1]*

**Value of the data**

• The data can be used for clarifying the impact of paracrine effects on renal parenchyma in terms of cell death.
• The data can also help for understanding how the cytokines could be used for monitoring the effects of the injected stem cells in a setting of pathologies, other than in CRSII.
• The data can be used to understand the effects of pluripotent cells injection in a setting of different pathologies.
• The data can be useful to other researchers to better understand the pathophysiology of CRSII.

## 1 Data

**Fig. 1:**
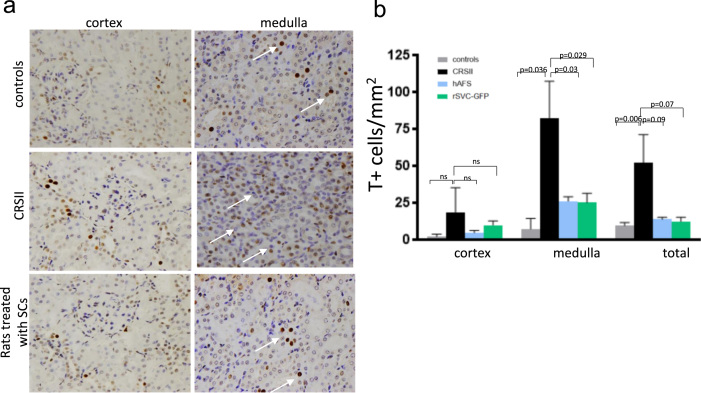
Cell death assay. (a) Representative histological images of controls, CRSII, hASF and rSVC-GFP rats made with the TUNEL technique, in cortex and medulla area of kidney. White arrows indicate TUNEL-positive cells identified by red-brown nuclei. Note as TUNEL-positive cells are mainly located in the medulla area of kidney. Original magnification 32×; (b) graph depicted the degree of apoptosis (TUNEL-positive cells, T+) quantified by TUNEL assay in kidney. In CRSII rats, cell death was significantly increased especially in the medulla area. For all groups of rats, cortex seems not to be affected by apoptosis. Cell-treated rats showed a significantly decrease of TUNEL+ nuclei in medulla area (*p* = 0.03 hAFS and *p* = 0.029 rSVC-GFP vs CRSII rats).

**Fig. 2:**
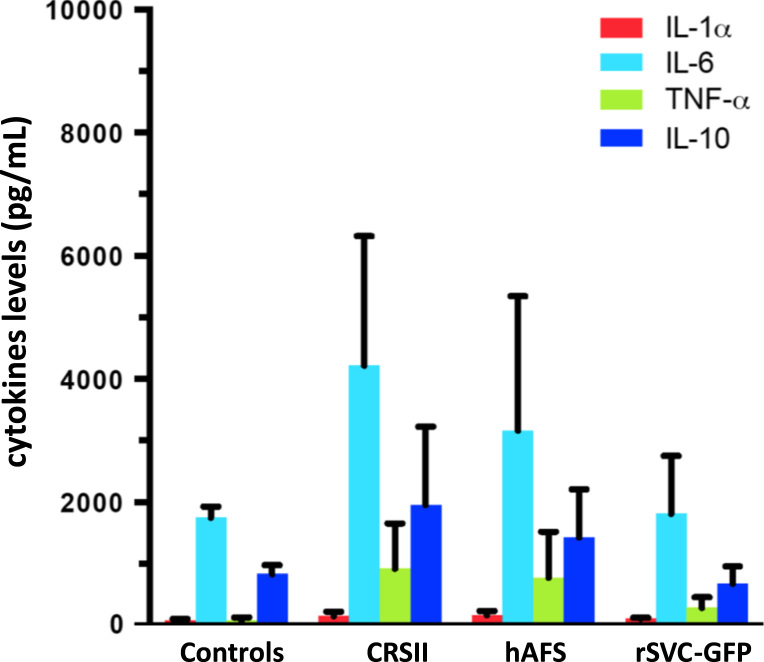
Circulating cytokines milieu. IL-1alfa, IL-6, TNF-alfa and IL-10 expressed in pg/mL in the four groups: hAFS, rSVC-GFP, CRSII, and control rats.

Data are presented in Table 1, Fig. 1, and Fig. 2.

**Table 1:** Kidney damage apoptosis.

	Cortex	Medulla	Total
Controls	2.481 ± 1.66^a^	7.601 ± 7.11^d^	9.858 ± 2.1^g^
CRSII	18.93 ± 16.51^a^^,^^b^^,^^c^	82.58 ± 25.01^d^^,^^e^^,^^f^	52.26 ± 19.28^g^, ^h^, ^i^
hAFS	5.01 ± 1.64^b^	26.4 ± 3.01^e^	14.07 ± 1.38^h^
rSVC-GFP	10.31 ± 2.67^c^	25.46 ± 6.35^f^	12.67 ± 2.96^i^

CRSII, cardiorenal syndrome type II rats; hAFS, c-Kit–selected human amniotic fluid stem cells; rSVC-GFP, rats vascular progenitor cells.

a *p* = 0.4 (CRSII vs controls)

b *p* = 0.7 (CRSII vs hAFS)

c *p* = 0.5 (CRSII vs rSVC-GFP)

d *p* = 0.03 (CRSII vs controls)

e *p* = 0.03 (CRSII vs hAFS)

f *p* = 0.0029 (CRSII vs rSVC-GFP)

g *p* = 0.006 (CRSII vs controls)

h *p* = 0.09 (CRSII vs hAFS)

i *p* = 0.07 (CRSII vs rSVC-GFP)

Table 1 reports numbers of apoptotic nuclei in all groups of experimental setting.

Histological images in Fig. 1a depict positive cells-nuclei, detected by cell-death TUNEL technique, showing that TUNEL-positive cells are mainly located in the medulla of kidney.

In Fig. 1b, no difference in apoptosis count in the cortex of the treated animals compared to CRSII group was detected.

Fig. 2 reports values of serum circulating cytokines in this experimental model of CRSII. Graph shows the cytokines modification in CRSII animal after both hAFS or rSVC-GFP cells injection.

## 2 Experimental design, materials and methods

For material and methods in details refer to Vescovo et al. [1].

### 2.1 Model of cardiorenal syndrome type II

Right sided heart failure (RHF) was induced in male Sprague-Dawley rats, weighting 90–100 g, by injecting intraperitoneally 30 mg/kg of monocrotaline (MCT) according to Vescovo et al. [1], [2], [3], [4], [5], [6], [7], [8], [9], [10], [11], [12].

MCT is a well established model of RHF which mimics the CHF syndrome in man [3], [4], [5], [6], [7], [8], [9], [10], [11], [12].

This experimental approach, after 4 weeks, produce a well established model of cardiorenal syndrome type II according to Angelini et al. [13].

### 2.2 Assessment of cell apoptosis in kidney tissue

in situ nick-end labeling (TUNEL) of fragmented DNA was performed on cryosections of the heart and kidney using an in situ cell death detection kit (POD; Boehringer Mannheim The number of positive nuclei was expressed as number of TUNEL-positive nuclei per square millimeter [1], [13], [14].

### 2.3 Bio-Plex multiplex cytokine assays

Serum samples were then analyzed through a Bio-Plex suspension array system (Bio-Rad, Milan, Italy) in order to quantify 9 cytokines: IL-1α, IL-1β, IL-2, IL-4, IL-6, IL-10, GM-CSF, IFN-γ and TNF-α, following the manufacturer׳s instructions [1], [13], [14].
