# Supplementary material for: Data on the stem cells paracrine effects on apoptosis and cytokine milieu in an experimental model of cardiorenal syndrome type II
Source: Data Brief. 2018 Nov 1;21:1430–4. doi: 10.1016/j.dib.2018.10.127 (PMC6234273; doi:10.1016/j.dib.2018.10.127)
Supplement: Supplementary file 1 — Supplementary material [file mmc1.pdf]

Author Agreement Form – International Journal of Cardiology

Manuscript Title: **Data on stem cells paracrine effects on apoptosis and cytokines milieu in an experimental model of cardio-renal syndrome type II**

List of all Authors: Giorgio Vescovo[1], Chiara Castellani[2], Marny Fedrigo[2], Grazia Maria Virzì[3,4], Giovanni Maria Vescovo[2], Regina Tavano[5], Michela Pozzobon[6], Annalisa Angelini[2]
Internal Medicine, S.Antonio Hospital[1], Dept. Cardiac, Thoracic,Vascular Sciences and Public Health[2], Department of Nephrology, Dialysis and Transplant, San Bortolo Hospital, Vicenza[3], IRRIV-International Renal Resarch Institute Vicenza[4], Dept. Biomedical Sciences[5], Dept. Women and Children Health[6] University of Padua, Italy

Corresponding Author: Annalisa Angelini

This statement is to certify that all authors have seen and approved the manuscript being submitted, have contributed significantly to the work, attest to the validity and legitimacy of the data and its interpretation, and agree to its submission to the *International Journal of Cardiology.*

We attest that the article is the Authors' original work, has not received prior publication and is not under consideration for publication elsewhere. We adhere to the statement of ethical publishing as appears in the International of Cardiology (citable as: Shewan LG, Rosano GMC, Henein MY, Coats AJS. A statement on ethical standards in publishing scientific articles in the International Journal of Cardiology family of journals. Int. J. Cardiol. 170 (2014) 253-254 DOI:10.1016/j.ijcard.2013.11).

On behalf of all Co-Authors, the corresponding Author shall bear full responsibility for the submission. Any changes to the list of authors, including changes in order, additions or removals will require the submission of a new author agreement form approved and signed by all the original and added submitting authors.

All authors are requested to disclose any actual or potential conflict of interest including any financial, personal or other relationships with other people or organizations within three years of beginning the submitted work that could inappropriately influence, or be perceived to influence, their work. If there are no conflicts of interest, the COI should read: "The authors report no relationships that could be construed as a conflict of interest".

Annalisa Angelini
